# Gut Microbiota Features Associated With *Campylobacter* Burden and Postnatal Linear Growth Deficits in a Peruvian Birth Cohort

**DOI:** 10.1093/cid/ciz906

**Published:** 2019-09-17

**Authors:** Saba Rouhani, Nicholas W Griffin, Pablo Peñataro Yori, Maribel Paredes Olortegui, Mery Siguas Salas, Dixner Rengifo Trigoso, Lawrence H Moulton, Eric R Houpt, Michael J Barratt, Margaret N Kosek, Jeffrey I Gordon

**Affiliations:** 1 Johns Hopkins Bloomberg School of Public Health, Baltimore, Maryland, USA; 2 Edison Family Center for Genome Sciences and Systems Biology, St. Louis, Missouri, USA; 3 Center for Gut Microbiome and Nutrition Research, Washington University School of Medicine, St. Louis, Missouri, USA; 4 Asociación Benéfica Proyectos en Informática Medicina y Salud, Iquitos, Peru; 5 University of Virginia, Charlottesville, Virginia, USA

**Keywords:** *Campylobacter*, enteropathy, microbiota, child growth

## Abstract

**Background:**

*Campylobacter* infection is associated with impaired growth of children, even in the absence of symptoms. To examine the underlying mechanisms, we evaluated associations between *Campylobacter* infection, linear growth, and fecal microbial community features in a prospective birth cohort of 271 children with a high burden of diarrhea and stunting in the Amazonian lowlands of Peru.

**Methods:**

*Campylobacter* was identified using a broadly reactive, genus-specific enzyme-linked immunosorbent assay. 16S rRNA-based analyses were used to identify bacterial taxa in fecal samples at ages 6, 12, 18, and 24 months (N = 928). Associations between infection, growth, and gut microbial community composition were investigated using multiple linear regression adjusting for within-child correlations, age, and breastfeeding. Indicator species analyses identified taxa specifically associated with *Campylobacter* burden.

**Results:**

Ninety-three percent (251) of children had *Campylobacter* present in asymptomatic fecal samples during the follow-up period. A 10% increase in the proportion of stools infected was associated with mean reductions of 0.02 length-for-age z scores (LAZ) at 3, 6, and 9 months thereafter (*P* < .01). We identified 13 bacterial taxa indicative of cumulative *Campylobacter* burden and 14 taxa significantly associated with high or low burden of enteroaggregative *Escherichia coli*, norovirus, or *Giardia*.

**Conclusions:**

*Campylobacter* infection is common in this cohort and associated with changes in microbial community composition. These results support the notion that disruptions to the fecal microbiota may help explain the observed effects of asymptomatic infections on growth in early life.

## Teaser

In this longitudinal cohort study, we demonstrate a high prevalence of asymptomatic *Campylobacter* infection among children aged 0–24 months, describe the effects of *Campylobacter* on gut microbial community composition, and link alterations in community composition to deficits in linear growth in early life.

**(See the Major Article by Rouhani et al on pages 989–99 and the Editorial Commentary by Colin Stine on pages 1008–9.)**

Linear growth faltering, or stunting, affects more than 160 million children annually and underlies a large proportion of childhood mortality and disability worldwide [1]. In low- and middle-income countries (LMICs), interactions between enteric infections, undernutrition, and diarrhea contribute to stunting [2, 3]. Evidence suggests that chronic or repeated enteropathogen infections without overt symptoms lead to intestinal inflammation, impaired gut barrier function, and blunted immune responses that, in turn, drive linear growth failure in the absence of diarrhea [4–8]. This may partly account for the persistence of stunting in areas where programs to prevent and treat clinical diarrhea and provide nutritional supplementation are well implemented [9–11].

*Campylobacter* may be an example of how enteropathogens can impair growth and immunity independently of clinical diarrhea [12]. Extensive literature links *Campylobacter* to Guillain-Barré syndrome, reactive arthritis, and irritable bowel diseases, demonstrating that infections can impact immunological tolerance and gut function beyond cessation of acute symptoms [13–18]. Data from large, multisite cohorts in LMICs reveal associations between *Campylobacter*, inflammation, gut permeability, and impaired linear growth [12, 19, 20], and studies have shown increased presence of *Campylobacteraceae* in the gut among undernourished children [21]. These observations are especially relevant given recent estimates of higher asymptomatic carriage than previously appreciated [22, 23].

In the present study, we test the hypothesis that *Campylobacter* is associated with specific changes in the developing gut microbiota. The rationale for exploring this issue comes from the increasing appreciation of the impact of *Campylobacter* on the intestinal environment and recent observations linking the gut microbiota to child growth. Birth cohorts have shown that assembly of the gut microbial community during early postnatal life exhibits shared features across individuals and geographic regions [24] and that this assemblage is critical for maturation of the gut mucosal immune system, barrier integrity, and other features of adaptive and innate immunity [25–27]. Moreover, studies have demonstrated impaired development of the gut microbiota in undernourished children in LMICs [28] and have used mouse models to provide evidence for a causal relationship between microbial composition and growth faltering [29].

In the current study, we use data from a birth cohort of 271 children to describe temporally structured associations between *Campylobacter* infection, fecal microbial diversity and composition, and linear growth. In our companion article presented in this issue of *Clinical Infectious Diseases*, we report that all-cause diarrhea provokes enduring changes to the gut microbial environment that, in turn, may impact subsequent growth acquisition and risk of illness. Here, we complement this analysis by determining whether asymptomatic *Campylobacter* infections incur independent effects on the gut microbiota in a manner that impacts intestinal integrity and child growth.

## METHODS

### Study Design

This study was conducted as part of the Etiology, Risk Factors and Interactions of Enteric Infections and Malnutrition and the Consequences for Child Health and Development Study, a birth cohort exploring enteropathy, nutrition, and child growth in 8 countries with high burdens of diarrhea and undernutrition. This study was conducted in the Amazonian lowlands near Iquitos, Peru. Details of demographic and biomedical data collection methods have been published previously [30–32]. Children were enrolled within 17 days of birth and followed for 24 months (n = 271). For surveillance of asymptomatic enteric infections, children contributed fecal samples and weight and length were measured monthly. Fieldworkers visited homes twice-weekly to record histories of illness, antibiotic use, breastfeeding, and dietary intake; additional specimens were collected during diarrheal episodes. Routine fecal samples obtained at postnatal months 6, 12, 18, and 24 were retrospectively selected for profiling of gut bacterial community composition. In total, 146 boys and 125 girls contributed 6096 months of observations, 6011 surveillance fecal samples, and 2440 diarrheal samples to the study.

### Diagnostics and Microbial Analyses

Swabs from fecal samples were placed into Cary-Blair transport medium and processed for culture on Campylobacter Agar base with Blaser’s supplement (Beckton Dickinson, Sparks, MD). Cultures were incubated at 42°C for 48 hours under microaerophilic conditions (5% oxygen, 10% carbon dioxide, and 85% nitrogen). Gram-negative colonies were tested for oxidase, catalase, and hippurate hydrolysis. Hippurate-positive *Campylobacter* strains were characterized as *Campylobacter jejuni* and hippurate-negative isolates as other *Campylobacter* species. *Campylobacter* testing directly on fecal samples stored at −70°C was done using a broadly reactive genus-specific ProSpecT enzyme-linked immunosorbent assay (ELISA) [33]. Details of diagnostic assays for >40 other enteropathogens are described elsewhere [34].

Procedures for isolating DNA frozen fecal samples, polymerase chain reaction–based amplification, and sequencing of the V4 region of bacterial 16S rDNA genes are published elsewhere [28, 29]. DNA sequences were oriented, trimmed to remove primer sequences, and paired using bbtools (37.02; https://sourceforge.net/projects/bbmap/). DADA2 (1.8.0) was used to remove chimeric sequences and identify and quantify amplicon sequence variants (ASVs) [35]. Taxonomic assignments were made using the RDP Naive Bayesian Classifier algorithm and the GreenGenes (13.8) training set [36]. Identified ASVs with taxonomic assignments are listed in Supplementary Table 1. ASVs were used to construct a neighbor-joining phylogenetic tree in phangorn (2.4.0) [37]. Metrics of community diversity and richness were estimated using the phyloseq [38] and picante (1.7) [39] packages. Three extreme outliers (>4 standard deviations for richness) and 2 samples taken during “exclusive breastfeeding” were removed before analysis.

### Definition of Covariates

Diarrhea was defined as ≥3 loose stools in a 24-hour period, with distinct episodes separated by 2 diarrhea-free days. Severity was measured using a community diarrheal assessment tool [40]. Asymptomatic infection was defined as detection of pathogens in surveillance stools in the absence of diarrhea or fever, and “pathogen pressure” was calculated as the mean number of enteropathogens per stool. Standard anthropometric and breastfeeding categories were based on World Health Organization (WHO) definitions [41, 42] and frequency of exposure to breastmilk was calculated as the cumulative mean number of feeds in the previous 24-hour period. Dietary diversity was measured as the number of WHO food groups (0–7) to which children were exposed [41].

We considered the number of distinct *Campylobacter-*positive diarrheal episodes and the cumulative proportion of surveillance stools with *Campylobacter* detected per child as exposure variables denoting symptomatic and asymptomatic infection, respectively. Diarrheal duration was calculated as the cumulative mean number of days per episode. The cumulative proportion of positive surveillance stools was divided into quartiles to compare the microbiota of children with the highest and lowest burdens. Persistent carriage was defined as 3 consecutive asymptomatic infections from monthly stools. We assessed gut bacterial diversity using Shannon’s and Simpson’s diversity indices; bacterial richness was assessed using the Chao1 index and phylogenetic diversity (see companion article for more details). We identified ASVs associated with high and low infection burden for *Campylobacter* and other enteropathogens with high prevalence in this population: *Giardia*, entero-aggregative *Escherichia coli* (EAEC), and norovirus.

### Statistical Analyses

To assess relationships between *Campylobacter* burden and specific ASVs, we conducted indicator species analysis (ISA). ISA identifies taxa indicative of particular habitats by calculating indicator species values for each ASV in each habitat [43], defined here as categorical groups of children with high or low enteropathogen burden. Each value is the product of the proportion of samples in 1 “habitat” (high or low burden) in which the ASV is detected and its mean relative abundance in that habitat, normalized by the sum of mean relative abundances across all habitat types. Indicator species values are bounded by 0 and 1, with 0 indicating the absence of an ASV from 1 group, and 1 indicating that an ASV occurs in every member of a group and only that group. Hypothesis tests were performed by permutation tests [43].

ISA was performed separately for each age category (6, 12, 18, and 24 months), comparing samples from children in low- and high-burden groups for *Campylobacter*, norovirus, EAEC, and *Giardia*. Within each ISA, *P* values were adjusted using the Benjamini-Hochberg method to control false discovery rates. Only ASVs found in at least 20% of the children in at least 1 enteropathogen burden group and having a mean percent abundance of 0.1% when present were included.

Associations between *Campylobacter*, length-for-age Z scores (LAZ), bacterial diversity and richness, and indicator ASVs were investigated using linear regression with generalized estimating equations and linear mixed-effects models to account for longitudinal sampling. We modeled asymptomatic and symptomatic *Campylobacter* infections as predictors of LAZ at the current time point and 3, 6, and 9 months thereafter; *Campylobacter* infections and diarrheal frequency, severity, and duration as predictors of intestinal diversity and richness; and the presence and abundance of indicator ASVs on LAZ at the time of sampling and 1 and 3 months thereafter. All models were adjusted for age, breastfeeding frequency and category, pathogen pressure, dietary diversity, days using antibiotics, and diarrheal frequency. Models of diversity metrics were also adjusted for the number of sequencing reads assigned to ASVs in order to control for differences in depth of sequencing. Models with “current” or “future” LAZ outcomes were additionally adjusted for LAZ at birth or current time point, respectively.

## RESULTS

### *Campylobacter* and Child Growth

**Figure 1.**
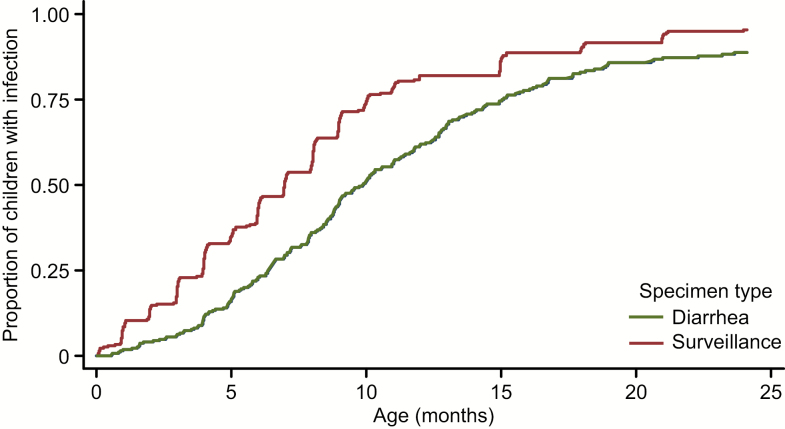
Time to first detection of *Campylobacter* by enzyme-linked immunosorbent assay in routine surveillance fecal samples and diarrheal specimens among infants aged 0–24 months in Santa Clara, Peru.

*Campylobacter* was detected by ELISA in 912 (22%) of surveillance stools and 758 (31%) of diarrheal specimens (Figure 1, Table 1). Only 22% (n = 364) of ELISA-positive samples were positive by culture-based methods, and this was consistent among surveillance (n = 197; 22%) and diarrheal samples (n = 167; 22%). *Campylobacter jejuni* accounted for nearly half of culture-positive diarrheal (45%) and asymptomatic fecal specimens (48%). By age 2 years, *Campylobacter* had been detected in ≥1 sample from each of 251 (93%) asymptomatic children. Mean time to first infection was 7.8 months, and detection increased with each month of age in the first year (odds ratio [OR], 1.21; *P* < .001). Fifty-five children (20.3%) experienced persistent infections. Of 260 children experiencing diarrhea, 221 (85%) had >1 *Campylobacter*-positive episode. The majority (n = 169; 64%) had *Campylobacter*-positive surveillance stools prior to clinical symptoms. Mean age of first *Campylobacter*-diarrhea was 9.3 months (Figure 1), with a mean of 2.6 episodes during the study. Twenty-seven percent of episodes were treated with antibiotics. Children who were exclusively or predominantly breastfed had significantly reduced odds of *Campylobacter*-diarrhea (OR, 0.39; *P* < .001) and asymptomatic detections (OR, 0.48; *P* < .001) after accounting for age.

**Table 1.** *Campylobacter* Infections Among 271 Children Aged 0–24 Months in the Peruvian Birth Cohort

	Age, months			
*Campylobacter* in the Population	6	12	18	24
% of children (n) ever infected	46.2 (115)	82.3 (181)	91.6 (185)	94.7 (178)
% of children (n) experiencing *Campylobacter*-positive diarrhea	22.5 (56)	60.0 (132)	82.2 (166)	88.3 (166)
Mean (range) of *Campylobacter-*positive diarrheal episodes by enzyme-linked immunosorbent assay	0.26 (0–4)	0.97 (0–4)	1.9 (0–11)	2.6 (0–15)
Mean (SD) proportion of surveillance stools per child with *Campylobacter* detected	12.6 (17.4)	19.2 (16.9)	21.3 (15.7)	22.5 (15.6)
Mean (SD) proportion of diarrheal samples collected from each child with *Campylobacter* detected	20.5 (31.4)	30.3 (21.9)	32.6 (25.5)	33.2 (24.4)

Abbreviation: SD, standard deviation.

By 24 months, 181 (66.8%) of children were stunted (LAZ < −2), and 22.1% were severely stunted (LAZ < −3) on ≥1 visit. The point prevalence of stunting at 24 months was 40%. *Campylobacter* infections were negatively associated with linear growth. An increase of 10% in the proportion of surveillance stools with *Campylobacter* was associated with a 0.02 reduction in LAZ at 3, 6, and 9 months thereafter (β = −0.02; *P* < .01 across all time points). Controlling for LAZ at birth, each episode of *Campylobacter*-diarrhea was associated with a reduction of 0.03 in current LAZ (95% confidence interval [CI], −0.04 to −0.01; *P* = .002). *Campylobacter*-positive diarrheal episodes were not predictive of subsequent LAZ scores.

### *Campylobacter* and the Gut Microbiota

There were no significant associations between fecal bacterial diversity and richness and frequency, duration, or severity of *Campylobacter-*positive diarrhea separately from all-cause diarrhea. In contrast, asymptomatic *Campylobacter* detections were significantly positively associated with all bacterial diversity and richness measures (Table 2).

**Table 2.** Evidence of Independent Associations Between *Campylobacter* Infections and Community Bacterial Diversity and Richness

Predictor Variable	Mean Change in Gut Microbial Diversity—β (95% Confidence Interval); Backtransformed β			
	Shannon’s Diversity	Simpson’s Diversity	Chao1 Index	Phylogenetic Diversity
Asymptomatic infection (per 10% increase in proportion of *Campylobacter*-positive fecal samples)^a^	0.048 (.023 to .072); 0.050***	0.069 (.031 to .107)***	0.035 (.016 to .053); 3.511***	0.041 (.016 to .066); 0.323**
No. of episodes of *Campylobacter*-positive diarrhea^b^	−0.012 (−.042 to .017); −0.013	−0.027 (−.07 to .015)	−0.001 (−.022 to .02); −0.07	0.011 (−.024 to .046); 0.088
Mean severity of *Campylobacter*-positive diarrhea^c^	−0.016 (−.054 to .022); −0.015	−0.017 (−.062 to .029)	−0.003 (−.027 to .022); −0.278	−0.009 (−.051 to .033); −0.068
Mean duration of *Campylobacter*-positive diarrhea^d^	−0.025 (−.056 to .006); −0.023	−0.022 (−.06 to .017)	−0.008 (−.029 to .012); −0.828	−0.025 (−.058 to .008); −0.189

Models were adjusted for age, breastfeeding frequency and category, pathogen pressure excluding *Campylobacter*, dietary diversity, antibiotic exposure, and the number of amplicon sequence variant–assignable DNA reads per sample. Coefficients for Shannon’s diversity and phylogenetic diversity are presented as the change in standard deviations and then backtransformed. For Simpson’s diversity, coefficients represent changes in logit-transformed values without backtransformations. For the Chao1 index, coefficients are in log-transformed units, and the backtransformed units represent percent change.

^a^Model adjusted for the proportion of surveillance stools infected with other enteropathogens.

^b^Model adjusted for the number of *Campylobacter*-negative diarrheal episodes experienced.

^c^Model adjusted for the mean severity.

^d^Model adjusted for the duration of *Campylobacter*-negative diarrhea to assess whether *Campylobacter* infection and diarrhea were independently associated with microbial diversity metrics.

**, *P* < .01; ***, *P* < .001.

**Figure 2.**
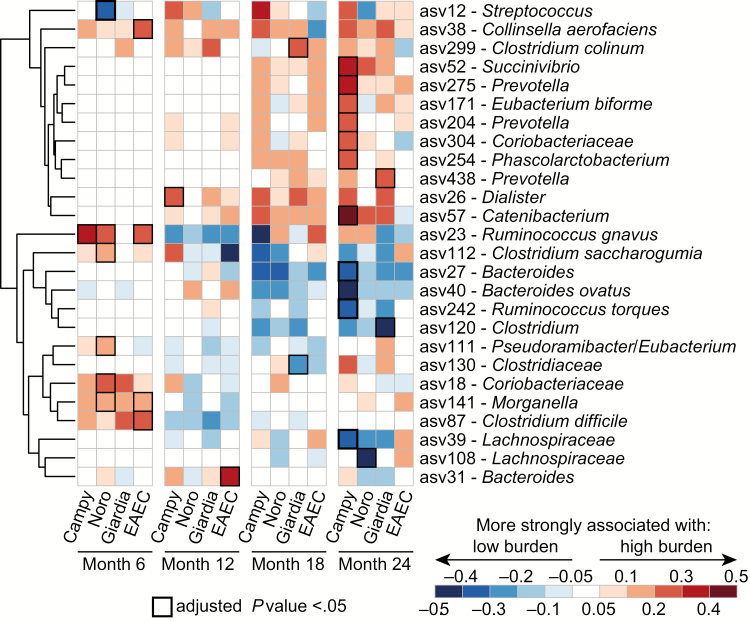
Associations between amplicon sequence variants (ASVs) and high vs low burden of enteropathogens at aged 6, 12, 18, and 24 months. The heat map shows the differences between indicator species values for high- and low-burden groups of *Campylobacter*, *Giardia*, EAEC, and norovirus. Red values indicate ASVs more associated with high burden, and blue values indicate ASVs more associated with low burden. Significant associations (adjusted *P* < .05) are indicated by boxes outlined in black. Abbreviations: Campy, *Campylobacter*; EAEC, entero-aggregative *Escherichia coli*; Noro, norovirus.

Thirteen ASVs were indicators of either high or low *Campylobacter* burden at 6, 12, or 24 months (Figure 2, Table 3, Supplementary Table 2). Nine of these ASVs were indicative of high *Campylobacter* burden, including *Ruminococcus gnavus* (ASV23) at month 6, a member of the genus *Dialister* (ASV26) at month 12, and ASVs assigned to *Prevotella* (ASV204 and ASV275), *Succinivibrio* (ASV52), *Catenibacterium* (ASV57), *Phascolarctobacterium* (ASV254), *Coriobacteriaceae* (ASV304), and *Eubacterium biforme* (ASV171) at 24 months. Four ASVs were indicative of low *Campylobacter* burden at month 24: *Bacteroides ovatus* (ASV40), *Ruminococcus toraues* (ASV242), members of *Bacteroides* (ASV27), and *Lachnospiraceae* (ASV39).

**Table 3.** Bacterial Amplicon Sequence Variants Significantly Associated With Low and High Asymptomatic Enteropathogen Burden at Aged 6, 12, 18, and 24 Months

Age (months)	Amplicon Sequence Variant Number	Taxonomy	Enteropathogen	Indicator Species Value		Fidelity (Frequency of Detection)		Specificity (Normalized Mean Relative Abundance)		Associated Burden Group	Adjusted *P* Value
				High	Low	High	Low	High	Low		
6	23	*Ruminococcus gnavus*	*Campylobacter*	0.333	0.032	0.415	0.163	0.802	0.198	High	.013
12	26	*Dialister*	*Campylobacter*	0.288	0.004	0.305	0.075	0.943	0.057	High	.012
24	304	*Coriobacteriaceae*	*Campylobacter*	0.222	0.003	0.240	0.039	0.926	0.074	High	.044
24	204	*Prevotella*	*Campylobacter*	0.270	0.004	0.300	0.039	0.900	0.100	High	.036
24	254	*Phascolarctobacterium*	*Campylobacter*	0.286	0.016	0.340	0.098	0.840	0.160	High	.044
24	171	*Eubacterium biforme*	*Campylobacter*	0.289	0.004	0.320	0.039	0.902	0.098	High	.013
24	275	*Prevotella*	*Campylobacter*	0.320	0.000	0.320	0.000	1.000	0.000	High	.006
24	52	*Succinivibrio*	*Campylobacter*	0.353	0.002	0.360	0.118	0.980	0.020	High	.036
24	57	*Catenibacterium*	*Campylobacter*	0.535	0.066	0.720	0.255	0.743	0.257	High	.006
24	242	*Ruminococcus torques*	*Campylobacter*	0.002	0.337	0.040	0.353	0.044	0.956	Low	.006
24	27	*Bacteroides*	*Campylobacter*	0.032	0.430	0.260	0.490	0.123	0.877	Low	.044
24	40	*Bacteroides ovatus*	*Campylobacter*	0.057	0.494	0.360	0.588	0.159	0.841	Low	.036
24	39	*Lachnospiraceae*	*Campylobacter*	0.251	0.646	0.800	0.941	0.313	0.687	Low	.040
6	141	*Morganella*	EAEC	0.190	0.018	0.240	0.085	0.791	0.209	High	.027
6	38	*Collinsella aerofaciens*	EAEC	0.234	0.006	0.260	0.062	0.900	0.100	High	.009
6	87	*Clostridium difficile*	EAEC	0.251	0.032	0.320	0.147	0.784	0.216	High	.023
6	23	*Ruminococcus gnavus*	EAEC	0.326	0.042	0.420	0.186	0.776	0.224	High	.023
12	31	*Bacteroides*	EAEC	0.330	0.018	0.404	0.098	0.817	0.183	High	.024
18	299	*Clostridium colinum*	*Giardia*	0.278	0.000	0.278	0.000	1.000	0.000	High	.013
18	130	*Clostridiaceae*	*Giardia*	0.000	0.242	0.019	0.243	0.005	0.995	Low	.019
24	438	*Prevotella*	*Giardia*	0.217	0.000	0.217	0.000	1.000	0.000	High	.017
24	120	*Clostridium*	*Giardia*	0.042	0.518	0.239	0.630	0.177	0.823	Low	.017
6	141	*Morganella*	Norovirus	0.185	0.030	0.273	0.093	0.679	0.321	High	.026
6	111	*Pseudoramibacter*/*Eubacterium*	Norovirus	0.208	0.025	0.258	0.132	0.809	0.191	High	.028
6	112	*Clostridium saccharogumia*	Norovirus	0.210	0.033	0.273	0.143	0.771	0.229	High	.026
6	23	*Ruminococcus gnavus*	Norovirus	0.261	0.049	0.348	0.192	0.748	0.252	High	.028
6	18	*Coriobacteriaceae*	Norovirus	0.336	0.101	0.485	0.330	0.694	0.306	High	.022
6	12	*Streptococcus*	Norovirus	0.334	0.645	0.970	0.984	0.344	0.656	Low	.017
24	108	*Lachnospiraceae*	Norovirus	0.057	0.482	0.292	0.600	0.197	0.803	Low	.034

Species indicative of high and low burden are shown in red and green, respectively. Expanded results with associations with adjusted *P* values < .1 are shown in Supplementary Table 1.

Abbreviation: EAEC, entero-aggregative *Escherichia coli.*

Several additional ASVs had indicator species values with adjusted *P* < .1, including 1 assigned to *Streptococcus* (ASV69; Supplementary Table 2), which was marginally indicative of low burden of *Campylobacter* at 6 months. A query of the GenBank 16S rDNA databank revealed that this ASV shares 100% sequence identity with *Streptococcus lactarius*, which has been isolated from the breast milk of healthy mothers [44]. The most abundant ASV assigned to the genus *Bifidobacterium* (ASV1) had a marginally significant association with low *Campylobacter* burden at 6 months (Supplementary Table 1). This ASV was detected in 97%–98% of the children in both high and low *Campylobacter* burden groups; its moderate association with low *Campylobacter* burden reflects higher relative abundance in that group. This ASV shares perfect sequence identity with published 16S rDNA reference sequences of constituents of the early developing gut microbiota during breastfeeding (*Bifidobacterium longum* subsp. *infantis*, *B. longum*, *Bifidobacterium brev* [24]).

Comparing the indicator species of high and low burden for *Campylobacter* with those for other enteropathogens revealed remarkably little overlap. Indicator species were not shared across high- and low-burden groups of the 4 enteropathogens assessed, with few exceptions. At age 6 months, *R. gnavus* (ASV23) was a high-burden indicator for *Campylobacter*, EAEC, and norovirus; *Morganella* (ASV141) was an indicator for high burden of EAEC and norovirus (but not *Campylobacter*; Figure 2, Table 3); and the *Streptococcus* ASV69 marginally associated with *Campylobacter* was also marginally associated with norovirus and EAEC.

Overall, 26 ASVs had significant indicator species values for low or high burden of at least 1 enteropathogen. As shown in Figure 2, despite the low degree of sharing of particular indicator ASVs, groups of ASVs behave similarly across enteropathogen burden and age groups. Hierarchical clustering divides these organisms into 2 major clusters, 1 predominated by ASVs indicative of high enteropathogen burden at 18 and 24 months and a second composed of ASVs indicative of high enteropathogen burden at 6 months and low burden thereafter. Moreover, differences between indicator species values reveal correlations within each age bin, suggesting that several ASVs correlated with high or low burden for multiple enteropathogens, even if their indicator species values were only statistically significant for 1. For example, at 6 months, the differences between indicator species values for high and low *Campylobacter* burden are correlated with those for norovirus (*r* = 0.67, *P* = .001), *Giardia* (*r* = 0.60, *P* = .005), and EAEC (*r* = 0.84, *P* < .001). At 24 months, the differences in indicator species values for high and low *Campylobacter* burden groups were correlated with those for norovirus (*r* = 0.55, *P* = .004) and *Giardia* (*r* = *0.80*, *P* < .001) but not for EAEC (*r* = *0.06*, *P* = .759).

The presence and abundance of each ASV listed in Table 3 were modeled as predictors of LAZ at the time of sampling and 1 month and 3 months thereafter across the entire 2-year sampling period, as well as separately at 6, 12, 18, and 24 months. After correction for false discovery rates, no single indicator ASV exhibited a statistically significant association with LAZ. However, in a linear model adjusting for LAZ at birth and sequencing depth, the aggregate number of ASVs indicative of high *Campylobacter* burden (Figure 2) was significantly associated with LAZ; specifically, each additional indicator of high *Campylobacter* burden detected in a sample at 24 months was associated with a reduction of 0.08 in LAZ score (*P* = .021).

## DISCUSSION

This study provides evidence for the hypothesis that asymptomatic *Campylobacter* burden is associated with changes to the gut microbial community that, in turn, may impact child growth in a setting of high enteric disease burden and stunting. Our concurrent work in the same Peruvian cohort demonstrates that all-cause diarrhea was associated with reductions in bacterial diversity and richness, that children with the most severe stunting profiles experience the greatest perturbations and longest recovery times following an episode of diarrhea, and that these perturbations are predictive of increased diarrhea, potentially leading to further loss of growth potential. In the present study, we provide evidence that *Campylobacter* burden is associated with linear growth faltering and changes to the gut microbial community in childhood in the absence of diarrhea. These observations suggest that alterations to gut microbiota associated with *Campylobacter* are independent from diarrhea-related purging and accelerated transit of upper gut flora. These results lend further evidence to the emerging hypothesis that asymptomatic *Campylobacter* infections contribute to childhood growth faltering in LMICs, possibly through alterations to the developing gut microbiota.

The development of the human gut microbiota in the first 2 years of life follows a path defined by several shared features. Increasingly, deviations from this normal developmental program have been associated with childhood growth deficits. Our observation that asymptomatic *Campylobacter* infections are associated with increased diversity among children aged 0–2 years, independent of other enteropathogens, may reflect a disruption of that developmental process. Indeed, breast milk exerts a suppressive effect on diversity in the infant gut, and limited diversity in early life may denote a healthy predominance of bacteria that metabolize breast milk and confer resilience to external insult [45–48]. This is consistent with the protective effects of exclusive or predominant breastfeeding against *Campylobacter* reported in this cohort [12]. Here, we also observed associations between low *Campylobacter* burden and a *Streptococcus* ASV that is found in breast milk, together with an ASV comprising several Bifidobacteria, including *B. longum* subs. *infantis* (*B. infantis). Bifidobacterium infantis* contains genes involved in the uptake and metabolism of human milk oligosaccharides and has an important role in healthy gut community assembly in early childhood [24, 49]. There were additional strong correlations between indicator ASVs and high burden of enteropathogens at 6 months. A number of these ASVs are typically not observed in the developing gut microbiota of healthy children until the second postnatal year [28]. This observation lends further support to the hypothesis that early deviations in microbiota assembly away from the lower diversity, predominantly breast milk–associated community may be associated with adverse health outcomes, including infection with enteropathogens.

In summary, we report changes to gut microbial populations associated with asymptomatic *Campylobacter* infections in a longitudinal study with a large sample size, generating testable hypotheses to improve control of a highly prevalent enteropathogen with adverse effects on growth in infancy and early childhood. A large proportion of infants in this setting are already exposed to *Campylobacter* by age 6 months. Our study is limited by the fact that we did not analyze samples prior to 6 months, precluding our ability to characterize the microbiota before infection. Results emphasize the need to address the question of cause and effect, that is, is *Campylobacter* uniquely equipped to disrupt community assembly or do specific community features predispose to *Campylobacter* invasion and perturbations in the normal functional maturation of the microbiota? Future studies should be extended to determine the generalizability of the results to populations in geographical settings with different cultural and dietary practices. Advancing these research objectives will help assess whether interventions designed to deliberately target the microbiota may be useful in mitigating the deleterious effects of *Campylobacter* on growth.

## Supplementary Data

Supplementary materials are available at *Clinical Infectious Diseases* online. Consisting of data provided by the authors to benefit the reader, the posted materials are not copyedited and are the sole responsibility of the authors, so questions or comments should be addressed to the corresponding author.

ciz906_suppl_Supplementary_Tables

## Notes

***Acknowledgments*.** The authors are grateful to the families of the birth cohort members described in this article for their participation and assistance and the communities of Santa Clara, Santo Tomas, and La Union. The authors thank Marty Meier, Su Deng, Justin Serugo, Kazi Ahsan, J. Hoisington-López, Monica McGrath, and Dennis Lang for technical assistance, and the Health Department of Loreto, Peru (DISA-Loreto). V4-16S rDNA sequencing datasets reported in this study are available through the European Nucleotide Archive (study accession number PRJEB28159).

***Disclaimer.*** Study funders had no role in study design, data collection, data analysis, interpretation, or manuscript preparation. The corresponding and senior authors had access to all data used in the analyses described and take final responsibility for publication decisions.

***Financial support.*** Funding was provided by the Bill & Melinda Gates Foundation; the Foundation for the National Institutes of Health; the Fogarty International Center; the Fisher Center for Environmental Infectious Diseases, Johns Hopkins University; the National Institute of Health (DK30292); and the Ken and Sherrilyn Fisher Foundation (to M. K.).

***Potential conflicts of interest.*** J. G. is a co-founder of Matatu, Inc, a company that characterizes the role of diet-by-microbiota interactions in animal health. All other authors report no potential conflicts. All authors have submitted the ICMJE Form for Disclosure of Potential Conflicts of Interest. Conflicts that the editors consider relevant to the content of the manuscript have been disclosed.
